# Spark-based parallel calculation of 3D fourier shell correlation for macromolecule structure local resolution estimation

**DOI:** 10.1186/s12859-020-03680-6

**Published:** 2020-09-17

**Authors:** Yongchun Lü, Xiangrui Zeng, Xinhui Tian, Xiao Shi, Hui Wang, Xiaohui Zheng, Xiaodong Liu, Xiaofang Zhao, Xin Gao, Min Xu

**Affiliations:** 1 Institute of Computing Technology of the Chinese Academy of Sciences, Beijing, China; 2 University of Chinese Academy of Sciences, Beijing, China; 3 Computational Biology Department, School of Computer Science, Carnegie Mellon University, Pittsburgh, USA; 4 King Abdullah University of Science and Technology (KAUST), Computational Bioscience Research Center (CBRC), Computer, Electrical and Mathematical Sciences and Engineering (CEMSE) Division, Thuwal, Saudi Arabia

**Keywords:** 3D local Fourier shell correlation, 3D local resolution map, Key-value data, Spark, 3D array partition

## Abstract

**Background:**

Resolution estimation is the main evaluation criteria for the reconstruction of macromolecular 3D structure in the field of cryoelectron microscopy (cryo-EM). At present, there are many methods to evaluate the 3D resolution for reconstructed macromolecular structures from Single Particle Analysis (SPA) in cryo-EM and subtomogram averaging (SA) in electron cryotomography (cryo-ET). As global methods, they measure the resolution of the structure as a whole, but they are inaccurate in detecting subtle local changes of reconstruction. In order to detect the subtle changes of reconstruction of SPA and SA, a few local resolution methods are proposed. The mainstream local resolution evaluation methods are based on local Fourier shell correlation (FSC), which is computationally intensive. However, the existing resolution evaluation methods are based on multi-threading implementation on a single computer with very poor scalability.

**Results:**

This paper proposes a new fine-grained 3D array partition method by key-value format in Spark. Our method first converts 3D images to key-value data (K-V). Then the K-V data is used for 3D array partitioning and data exchange in parallel. So Spark-based distributed parallel computing framework can solve the above scalability problem. In this distributed computing framework, all 3D local FSC tasks are simultaneously calculated across multiple nodes in a computer cluster. Through the calculation of experimental data, 3D local resolution evaluation algorithm based on Spark fine-grained 3D array partition has a magnitude change in computing speed compared with the mainstream FSC algorithm under the condition that the accuracy remains unchanged, and has better fault tolerance and scalability.

**Conclusions:**

In this paper, we proposed a K-V format based fine-grained 3D array partition method in Spark to parallel calculating 3D FSC for getting a 3D local resolution density map. 3D local resolution density map evaluates the three-dimensional density maps reconstructed from single particle analysis and subtomogram averaging. Our proposed method can significantly increase the speed of the 3D local resolution evaluation, which is important for the efficient detection of subtle variations among reconstructed macromolecular structures.

## Background

Macromolecular complexes are nano-machines involved in a wide range of biological cellular processes. The biological functions of macromolecular complexes are determined by their 3D structures. Therefore, it is important to know the structures of these macromolecule complexes to fully understand these cellular processes [1].

In recent years, electron cryomicroscopy (cryo-EM) has played an increasingly important role in 3D structure recovery of macromolecular complexes. 3D structures of biological macromolecules and complexes can be further studied to reveal their specific function, which may lead to major breakthroughs in biological cellular mechanisms, pharmacy and disease treatment, etc.

The single particle analysis (SPA) techniques in cryoelectron microscopy (cryo-EM) and subtomogram averaging (SA) techniques in electron cryotomography (cryo-ET) are widely applied to obtain 3D density maps of macromolecular complexes [2, 3]. The quality of reconstructed 3D density maps needs to be evaluated normally in terms of the structural resolution. The resolution can be directly utilized to interpret the extent of the structural details in the density map. A number of different methods have been proposed on resolution evaluation, among which popular ones are Fourier shell correlation (FSC) [4–6], spectral signal-to-noise ratio (SSNR) [7], R measure [8], likelihood-ratio hypothesis testing (ResMap) [9], and monogenic signal transformation (MonoRes) [10].

In this paper we focus on FSC, the most widely used resolution evaluation method [6, 11, 12]. It is normally employed in the SPA and SA for resolution evaluation. The golden standard procedure is to divide the data into two independent halves and recover separately. Then the normalized cross correlation between the two results with different shell in Fourier space is calculated.

In such resolution estimation, all particles are considered structurally homogeneous under ideal conditions in the SPA technique and SA technique, save for different orientations and positions, so the FSC can measure resolution of density maps of reconstruction. However, in practice, particles of a macromolecular complex can often be structurally heterogeneous [13–15]. The traditional global FSC measure cannot be used to accurately detect the subtle structural variations in the reconstructions of SPA and SA. In order to evaluate local structural heterogeneity, a local resolution evaluation has been proposed [6, 9, 10]. The first 3D local resolution evaluation method is blocres [6], which employed windowed FSC to calculate local density maps. The method utilizes a density map of windows to calculate corresponding FSC value. The blocres method extends 2D FSC evaluation to 3D local FSC evaluation. Implementation of blocres utilizes Open Multi-Processing (OpenMP) to parallelize calculation on a single compute node.

The existing methods of FSC-based local resolution evaluation are single-node multi-threaded implementation. Part of the reason is that single-node multi-threading implementation can satisfy local resolution evaluation, but multi-threaded implementation often considers thread security and is not scalable. Although it is relatively accurate to obtain 3D local resolution map by calculating 3D local FSC, the computations are enormous, mainly due to a large number of coupled 3D small windows calculated. For volume density map of 300×300×300 voxel with 0.982Å resolution, computational time is 123.5 minutes for the most accurate algorithm, blocres, at criterion of FSC 0.5 [10]. Hence, it is necessary to develop a distributed parallel method of calculating 3D local FSC for 3D local resolution evaluation. Specifically, by applying distributed computing algorithms, one can quickly chalk up 3D local resolution map through parallel computing on a cluster, not just on one compute node. Nevertheless, the main challenge for distributed algorithms design and development is how to effectively divide a 3D array (volume density map) into 3D sub-arrays (sub-volume density map), distribute them on the computer cluster, and result of each pair 3D sub-array’s calculation is not influenced by other threads.

In this paper, we present a Spark-based parallel method to calculate 3D local FSC for 3D local resolution estimation. Our method converses 3D images to K-V data. It then performs fine-grained 3D array partition by K-V format of Spark in memory. This method utilizes Spark [16] to implement parallel and distributed computation on a cluster. Our method can obtain the 3D local resolution map significantly faster than blocres method without losing accuracy. With a sufficiently large number of compute nodes, even in a few seconds, the 3D local resolution map can be computed. In order to divide 3D array into plenty of 3D sub-arrays for parallel computation, we propose a K-V format based basic fine-grained 3D array partition method in Spark. To further reduce memory space, data shuffling and transfer, we propose a K-V format based optimized fine-grained 3D array partition method in Spark. The 3D density map is essentially a 3D gray image represented as a 3D array. The 3D array partition method can be easily extended to other 3D array operations. A detailed comparison of different local resolution methods are performed to illustrate the advantages of our Spark-based 3D local resolution algorithm.

Our Spark-based 3D local resolution estimation algorithm can achieve completely automatic calculation of 3D local FSC values and resolution with suitable window size and specified FSC threshold, and is significantly faster than the blocres algorithm. In addition, our algorithm is flexible and can be run on a single compute node with specified number of cores.

## Related work

The 3D local resolution evaluation methods [6, 9, 10] can inspect the subtle changes during reconstruction of particles in SPA and SA, however, these methods can only be used on a single compute node (Table 1), and cannot quickly compute the 3D local resolution results. Some of these methods are based on the FSC method, while others are based on hypothesis testing.

**Table 1:** Introduction of different 3D local resolution evaluation methods

Methods	Characteristics	Is distributed?
blocres	based on FSC, window sampling	no
ResMap	based on 3D sinusodial wave, hypothesis testing	no
MonoRes	based on monogenic signal, hypothesis testing	no

### FSC-based methods for 3D local resolution estimation

**blocres algorithm**: For the blocres [6] algorithm, the moving window is used to traverse the half-map respectively, and the area of two 3D windows is calculated using FSC to gain local resolution. Finally, the local resolution is filled into the new 3D density map corresponding to coordinate, and the new 3D density map is the same size of half-maps. Because FSC measures are calculated without any transformation on the original density maps, blocres is the most accurate algorithm in calculating local resolution. In the blocres method, many windows will be generated, and it is very time-consuming to calculate these windows using FSC and acquire local resolution. This poses an issue when coupled with the fact that the blocres method runs on a single compute node.

### Hypothesis testing based methods for 3D local resolution estimation

Besides local FSC, another kind of local resolution evaluation is based on likelihood-ratio hypothesis testing of density map approximated by basis function versus noise or signal transformation, such as ResMap [9] and MonoRes [10].

**ResMap algorithm**: For ResMap [9] algorithm, the following conditions need to be followed: if a 3D local sinusoid of wavelength *λ* can be detected statistically higher than noise at this point, then there exists a *λ*-Å characteristic at this point in the volume. The likelihood ratio hypothesis testing of the local sinusoid curve and noise is employed to detect the feature when *P* value is 0.5. The local resolution at this point is the minimum *λ* that can detect the local sinusoid curve. A set of the second-order Hermite polynomials function expresses the local sinusoids of wavelength *λ*. However, the ResMap algorithm has some limits, such as the need for an initial step and running on single compute node.

**MonoRes algorithm**: MonoRes [10] is the intensity map of the signal, which is converted by the initial electron density map at that location, and also called monogenic magnitude map. The transformation of signal is Riesz transform (or Hibert transform of 1D signals). Riesz transform is a generalization of Hibert transform’s multidimensional (N-D), defined only for 1D signals. The map generated by MonoRes algorithm is the same size as the original map, where each voxel participates in the local resolution estimation. For each voxel of the mask volume, MonoRes algorithm use hypothesis testing to detect whether the local monogenic amplitude is greater than the 1 - *α* percentile of the monogenic amplitude in noise. MonoRes algorithm runs on a single compute node.

Since the hypothesis testing is used in ResMap and MonoRes algorithms, the density map approximated by transformation is faster in calculation, but still costs a few minutes for density maps of size 200^3^ voxels [10]. Generally, the density map approximated by transformation is constrained with various factors, and one of the key factors is resolution of density map. Due to the low signal-to-noise ratio (SNR), using transformation to approximate density map of reconstruction cannot improve the local resolution estimation under low resolution conditions. The method of local resolution evaluation based on FSC directly employs two half-maps to calculate, and doesn’t need to approximate the density map using specific basis functions, so that the details of the reconstructed density map can be evaluated more accurately. Although the calculations of local resolution evaluation used in FSC are larger, these methods reflect the resolution of density map more objectively and are more suitable for the resolution evaluation of SA in cryo-ET.

### Distributed computation frameworks

Due to the low SNR of particles in SPA and SA, FSC is still widely applied [17] for 3D local resolution estimation to assess the details during reconstruction of SPA and SA. In order to gain 3D local resolution map faster, a distributed version of 3D local resolution evaluation algorithm is implemented. Currently, there exist many mainstream distributed computation frameworks, such as Message Passing Interface (MPI), Hadoop MapReduce and Apache Spark.

**Message Passing Interface (MPI)**: MPI [18] is a standardized portable messaging standard, which can be used in various parallel computing architectures. MPI defines the grammar and semantics of the library routines’ core, and is also a communication protocol of programming parallel computers, and styled for supporting point-by-point and collective communication. MPI is an application programming interface of messaging, together with the protocols and semantic specifications that its features must represent in any implementation. MPI has the features of high performance, scalability and portability. MPI remains the main model in high performance computing [19], but it is not easy to implement and has no fault-tolerant mechanism and redundancy mechanism.

**Hadoop MapReduce**: MapReduce [20] is a programming model. It is a related implementation of using parallel distributed algorithms to process and generate large data sets in a cluster. MapReduce programs consist of map processes that perform filtering and sorting, and reduce processes that perform summary operations. MapReduce combines the distributed serves, runs the different tasks in parallel, manages all communication and data transmission in a different system, and provides redundancy and fault tolerance [21, 22]. MapReduce processes K-V data. MapReduce framework handles data (e.g input and output data) from disk.

**Apache Spark**: Apache Spark [16] is a generic open source distributed cluster computing framework. Spark provides an interface to implement data parallelism and programming on a cluster. Spark is based on the resilient distributed datasets (RDD), which is a read-only multi-set data item distributed on a set of machines and maintained in a fault-tolerance manner. The Dataframe Application Programming Interface (API) is published as an abstraction over RDD, followed by the Dataset API. RDD technology remains the foundation of the Dataset API, and the operations of RDD rely on K-V data. Spark framework only deals with data in memory. Spark framework is better suited for fast data processing and iterative processing [23].

A 3D local resolution evaluation algorithm based on FSC needs a large number of sub-volumes to participate in calculation, which contains iterative processes [24, 25]. Hence, Spark framework is better suited for fast data processing and iterative processing. Because Spark is only a coarse-grained distributed framework, for a specific task such as local FSC computation, we need to design a K-V form based novel fine-grained 3D data partition and transformation algorithm for distribution parallel computing in Spark.

## Results and discussions

To inspect the performance of our Spark-based optimized 3D local resolution estimation algorithm, this method was applied to two experimental density maps, and compared with other methods in computing time and accuracy.

### Comparison of time cost between different methods of 3D local resolution evaluation

First, we compare time cost between our Spark-based optimized 3D local resolution estimation algorithm and blocres [6] algorithm. In order to objectively evaluate both algorithms, we followed the same parameter conditions with two experimental data sets respectively.

To gain 3D local resolution map faster, our Spark-based optimized 3D local resolution estimation method can extend compute node freely until all tasks can parallel work simultaneously. But blocres method only works under single compute node. For distinguishing time cost, we just employ four compute nodes and eight compute nodes for Spark parallel algorithm.

**Fig. 1:**
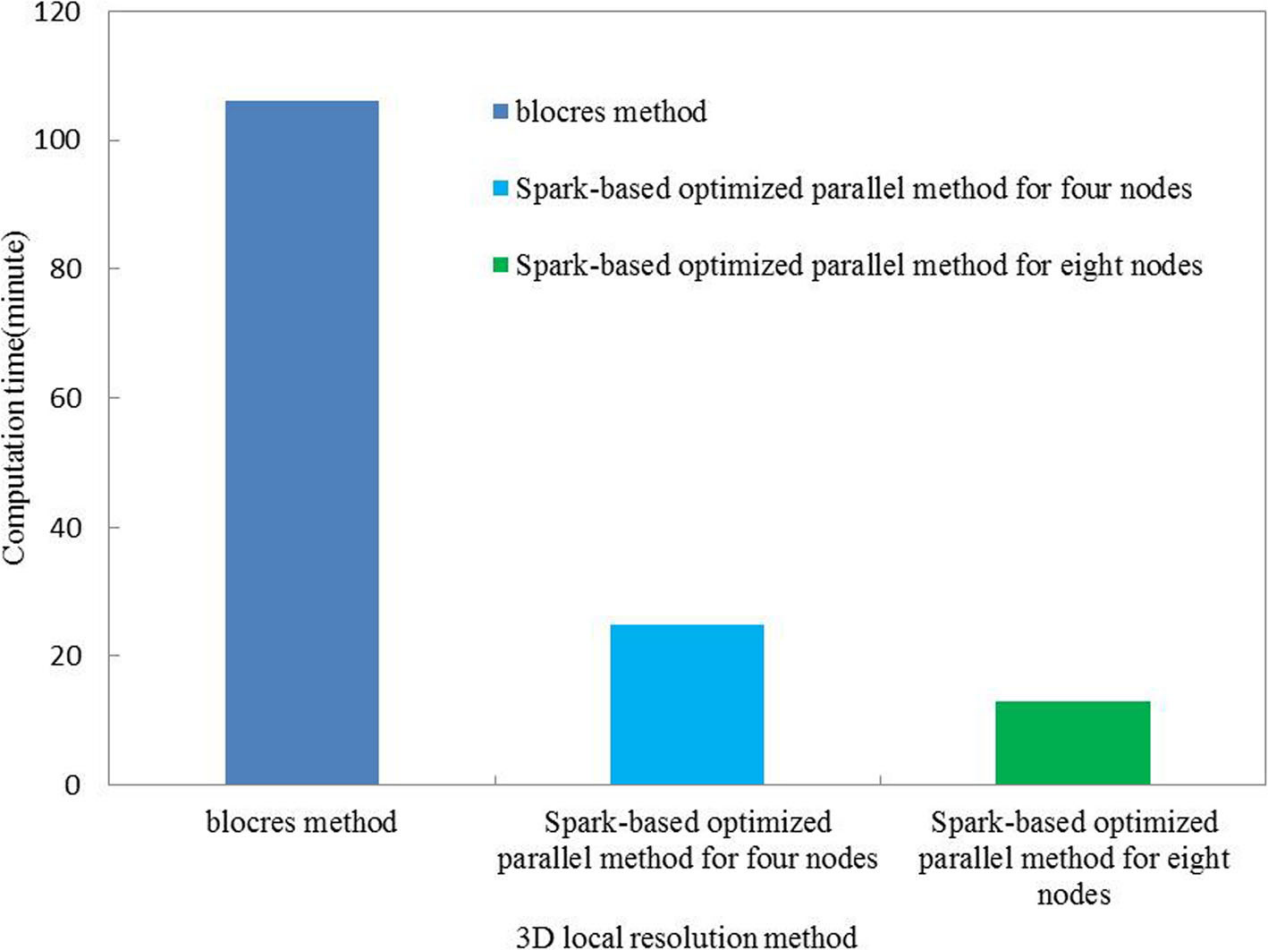
The time cost of Spark-based optimized 3D local resolution estimation method and blocres method

Under two 3D local resolution methods, we used windows size of 64^3^ and FSC of 0.143 criteria to traverse the half-maps (EMDB:EBD-3802), and calculated 3D local FSC and resolution respectively. From Fig. 1, blocres method consumed about 106 minutes for 3D local resolution estimation during two half map of the human TFIIH (EMDB: EMD-3802), however, our Spark-based optimized 3D local resolution estimation method only utilized 25 minutes and 13 minutes under the four and eight computing node respectively.

The above observations indicate that our Spark-based optimized 3D local resolution estimation method is significantly faster than the blocres method. Assuming more computing nodes, even virtual machine nodes, our Spark-based optimized 3D local resolution estimation method can perform all tasks simultaneously, whereas the blocres algorithm cannot be implemented.

### Comparison of accuracy between different method of 3D local resolution evaluation

In order to compare accuracy of 3D local resolution map resulting from blocres method and Spark-based optimized 3D local resolution estimation method, we tested two experimental data: one is half map from SPA, one is half map from SA.

**Fig. 2:**
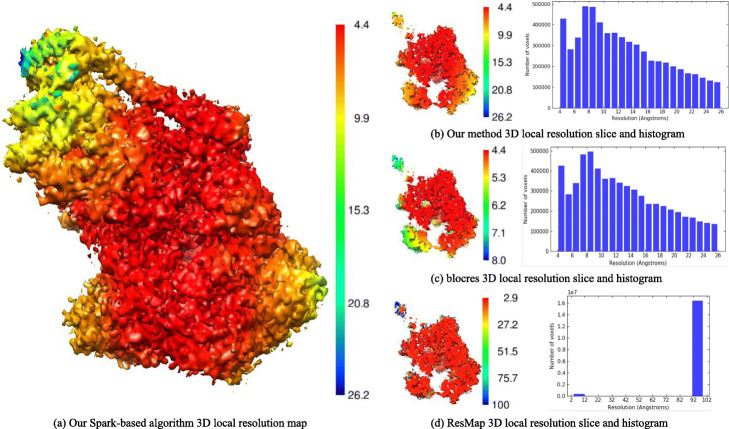
Spark-based 3D local resolution density map. Spark-based, blocres and ResMap slices, and histogram for experimental maps. **a** Spark-based 3D local resolution density map. **b** Spark-based method resolution slice and histogram for the cryo-EM structure of human TFIIH (EMDB: EMD-3802). **c** blocres method resolution slice and histogram for the cryo-EM structure of human TFIIH. **d** ResMap method resolution slice and histogram for the cryo-EM structure of human TFIIH

First, through applying two half-maps of cryo-EM structure of *β*-galactosidase, the 3D local resolution maps gained by our Spark-based optimized 3D local resolution estimation method and blocres method are shown in Fig. 2. Distribution by histogram, 3D local resolution estimation of Spark-based optimized 3D local resolution estimation method range from 4.4 to 26.2Å, and 3D local resolution estimation of blocres method range from 4.4 to 26.2Å. Because a large number of resolution values were 0Å in the edge of the volume, the median of 3D resolution of our Spark-based optimized 3D local resolution estimation method and blocres method were 0 and 0Å respectively. The color range of slice of our Spark-based optimized 3D local resolution estimation method and blocres method were a little different, which were 4.4 to 26.2Å and 4.4 to 8.0Å respectively. The color range of slice and histogram in our Spark-based optimized 3D local resolution estimation method was almost the same. The original resolution of cryo-EM reconstruction of human TFIIH published in [26] was 4.4Å. All methods were under the FSC of 0.143 criterion.

Through the histogram of our Spark-based optimized 3D local resolution estimation method and blocres method, we can found that the resolution value distribution of Spark-based optimized 3D local resolution estimation method was similar to that of the blocres method. So combining the histogram and slice of two methods, the two 3D local resolution maps were almost similar, and from the 3D local resolution maps, the detail of cryo-EM reconstruction of human TFIIH can be reflected. We also utilized ResMap method to obtain 3D local resolution map, which were colored by Chimera with the final reconstruction (EMDB: EDB-3802) in Fig. 2d, 3D local resolution estimation of ResMap method range from 2.9 to 100Å, the median of ResMap was 100Å. Although the histogram of our Spark-based optimized 3D local resolution estimation method and ResMap method was different, the color range of slice of our Spark-based optimized 3D local resolution estimation method was similar to that of ResMap method.

**Fig. 3:**
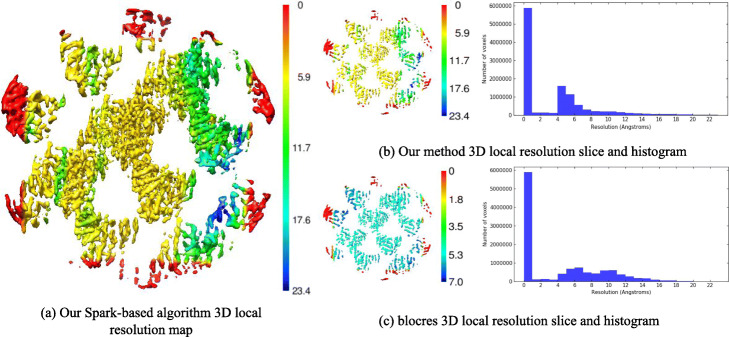
Spark-based 3D local resolution density map. Spark-based and blocres slices, and histogram for experimental maps. **a** Spark-based 3D local resolution density map. **b** Spark-based method resolution slice and histogram for SA of Sar1-Sec23-Sec24 (EMDB: EMD-0044). **c** blocres method resolution slice and histogram for SA of Sar1-Sec23-Sec24

Next, using two half-maps of SA of Sar1-Sec23-Sec24 (EMDB: EMD-0044), the 3D local resolution maps were obtained by our Spark-based optimized 3D local resolution estimation method and blocres method respectively (Fig. 3). Distribution by histogram, the 3D local resolution range estimated by our Spark-based optimized 3D local resolution estimation method and the blocres method was between 0 and 23.4Å with the median of 0.0Å. By using the tool Surface Color in Chimera, the color range of slice of Spark-based optimized 3D local resolution estimation method was 0 to 23.4Å, and the color range of slice of the blocres method was 0 to 7.0Å. Although the color range of two methods were a little different, the histograms of the two methods were almost the similar. The covered resolution of the SA of Sar1-Sec23-Sec24 was 4.9Å [27]. From the Fig. 3. The 3D local resolution map of our Spark-based optimized 3D local resolution estimation method achieved similar results compared with the 3D local resolution map of blocres method, and our Spark-based optimized 3D local resolution map had the same resolution on the boundary.

## Conclusion

In this paper, we focus on reducing the time cost of the local FSC calculation for macromolecule 3D structure local resolution estimation. The traditional 3D local resolution algorithm by calculating 3D FSC are computationally intensive and normally only execute under single compute node, therefore we consider distributed 3D local resolution estimation algorithm in order to significantly increase the speed of obtaining 3D local resolution map.

By analyzing the mainstream distributed architecture, we choose Spark architecture for efficient K-V data processing to design a distributed 3D local resolution evaluation algorithm. Specifically, Spark architecture is more suitable for iterative algorithm because K-V data operations are implemented in memory, which does not need frequent IO exchange. Also, Spark structure has strong extensibility and fault tolerance.

In this paper, we propose two kinds K-V format based fine-grained 3D array partition algorithms in Spark for computing 3D FSC in parallel. Compared with the K-V format based basic fine-grained 3D array partition algorithm, the K-V format based optimized fine-grained 3D array partition algorithm is more memory-saving, faster in calculation and more concise in expression. The K-V format based optimized fine-grained 3D array partition algorithm in Spark as the core of the Spark-based optimized 3D local resolution estimation method. Spark-based optimized 3D local resolution estimation method can quickly get the 3D local resolution map and evaluate the details of single particle analysis reconstruction in cryo-EM and average reconstruction of subtomogram in cryo-ET.

Through the calculation of experimental data, Spark-based optimized 3D local resolution estimation method are faster than blocres method to gain 3D local resolution map, but still lack some optional functions, such as filtering and mask etc. We plan to develop more full-featured algorithms in the future. Integrating graphics processing unit (GPU) into our current framework may further accelerate computation. Spark-based optimized 3D local resolution estimation method can be applied in cloud services and cluster environments. When users are computing 3D local resolution in a cloud server or cluster with multiple idle computing nodes, Spark-based optimized 3D local resolution estimation method can make full use of these computing nodes to quickly calculate 3D local resolution. Spark-based optimized 3D local resolution estimation method can also support on-line fast calculation of 3D local resolution and avoid the user’s online waiting time. In the future, we will optimize the scheduling algorithm, through analyzing the size of the three-dimensional volume submitted by the user, and reasonably allocate the calculation nodes, so as to quickly realize the calculation of the 3D local resolution.

## Methods

In this section, we first introduce the resolution, the 3D FSC, the 3D local FSC and 3D local resolution estimation method. Then, we describe two kinds of K-V format based fine-grained 3D array partition algorithms for 3D local resolution evaluation. We use Spark’s efficient K-V data batch processing platform to realize the distributed parallel computing of 3D local FSC. This algorithm extends the multithreading of a single computing node to the distributed parallel computing in spark. Finally, we depict the algorithm implementation and experimental datasets.

### The resolution and 3D fourier shell correlation

Resolution in the electron density expresses a measure of electron density resolvability. In cryo-EM, resolution is a comparison of frequency space correlation of two density maps of the data divided into two halves. Resolution is typically measured by the spatial frequency correlation function in cryo-EM.

The method of 3D FSC, initially proposed by Harauz and van Heel [5] in 1986, is frequently applied as quantitative evaluation of the reconstruction volume resolution in SPA and SA. The 3D FSC method mainly obeys the following process: two density maps of 3D volumes are reconstructed with one half data set in SPA or SA respectively, two 3D volumes are transformed from real space into Fourier space and form a series of shells, and then the corresponding two series of shells in Fourier space are calculated and gained normalization cross correlation coefficient. Hence, the 3D FSC effectively evaluates the resolution of reconstruction density maps with the normalization cross correlation coefficient between two series of shells, which are generated by 3D volumes in Fourier space.

The 3D FSC [6] between the two density maps in Fourier space, *F*_1_ and *F*_2_, is represented by

1 $$ {FSC}_{1,2}(s) = \frac{{\sum\nolimits}_{s_{i}\in{s}}F_{1}(s_{i}) \cdot F_{2}(s_{i})^{\star}}{\sqrt[]{{\sum\nolimits}_{s_{i}\in{s}}|F_{1}(s_{i})|^{2} \cdot \sum_{s_{i}\in{s}}|F_{2}(s_{i})|^{2}}}  $$

where *s*_*i*_ is corresponding a series of shells *s* in Fourier space, *F*(*s*_*i*_) is complex structure factor at radial frequency *s*_*i*_ in Fourier space, ${\sum \nolimits }_{s_{i}\in s}$ is summation over all Fourier space voxels *s*_*i*_ in shell s. *F*(*s*_*i*_)^⋆^ denotes a conjugate complex of *F*(*s*_*i*_).

The 3D FSC method extends the pristine two-dimensional Fourier Ring Correlation (FRC) algorithm [4], and obtains density map of 3D FSC to evaluate the resolution of reconstructed density map more visually and scientifically.

### 3D local fourier shell correlation and 3D local resolution map estimation

To better evaluate details of resolution of reconstructed density map from SPA in cryo-EM and SA in cryo-ET, the 3D local resolution evaluation method based on 3D FSC has been proposed in [6], which is implemented by 3D local FSC.

For given two half-maps (3D density maps), two small cube windows are used to traverse their respective 3D density maps. Each time a small cube window accesses the 3D density maps, the 3D density map of the corresponding region of the two small cube windows are calculated to generate 3D FSC value, and then are converted to the corresponding resolution value by specified FSC threshold and voxel value. The resolution value is filled into the corresponding center position of the small cube window in the new 3D volume, and the corresponding other position of the small cube window in the new 3D volume does not fill in the value, and then operate in turn until the small cube window ends traversing the 3D density map. In the new resolution 3D, the corresponding center position of traversing small cube window will be filled in the value in turn, so that the new resolution 3D (the size and the 3D density map are the same) generate discrete resolution values, and then 3D cube interpolation methods are used to interpolate coordinate positions without values in turn. When the new resolution 3D volume is filled completely with resolution values, this resolution 3D volume is called 3D local resolution map.

On the 3D local resolution map, the size of the accessed small cube window and the step size of traversal are the pivotal factors affecting the 3D local resolution map. If the size of the small cube window is too small, the calculation of smaller FSC threshold (e.g. FSC = 0.143) cannot be performed for local resolution estimation. Similarly, if the size of the small cube window is too large, 3D local resolution map will not be veritable. The step size of traversal also influences the 3D local resolution map. The larger the step size of the traversal, the fewer the 3D local resolution values to be calculated, which need to be interpolated in the new 3D resolution volume map and cannot veritably reflect the resolution situation. The smaller the step size of the traversal, the more the calculated 3D local resolution values, and thus this is closer to the real 3D local resolution map, However, the amount of computation is too large. As a result, it is highly prudent to determine the appropriate size of the small cube window and the step size of the traversal.

However, 3D local resolution evaluation method not only calculates 3D FSC in small windows, but also considers global resolution calibrating the average value of all local resolutions for accurate estimating local resolution. Hence, 3D local resolution evaluation method is influenced by windows side length, step of the traversal, threshold of the FSC, global resolution and so on.

### Spark-based parallel calculation of 3D local FSC for estimation of 3D local resolution

To significantly reduce the time used for computing 3D local FSC, we propose a Spark-based distributed 3D local resolution evaluation method with a parallel and distributed computing architecture.

In our 3D local resolution evaluation algorithm, the K-V format based fine-grained 3D array partition can facilitate the distributed parallel computing of 3D FSC, so as to obtain the 3D local resolution map quickly. As discussed in Distributed computation frameworks Section, Apache Spark has more advantages than MPI and MapReduce, processes K-V data in memory, owns redundancy mechanism and fault tolerant mechanism, and is suitable for high frequency data exchange and large iterative processing.

Based on Apache Spark distributed framework, we design two methods of fine-grained 3D array partition algorithms by K-V format in Spark. Two methods can calculate 3D local FSC in parallel, so as to acquire 3D local resolution estimation map faster. This algorithm can be applied not only to SA and SPA, but also to other 3D image operations.

**Fig. 4:**
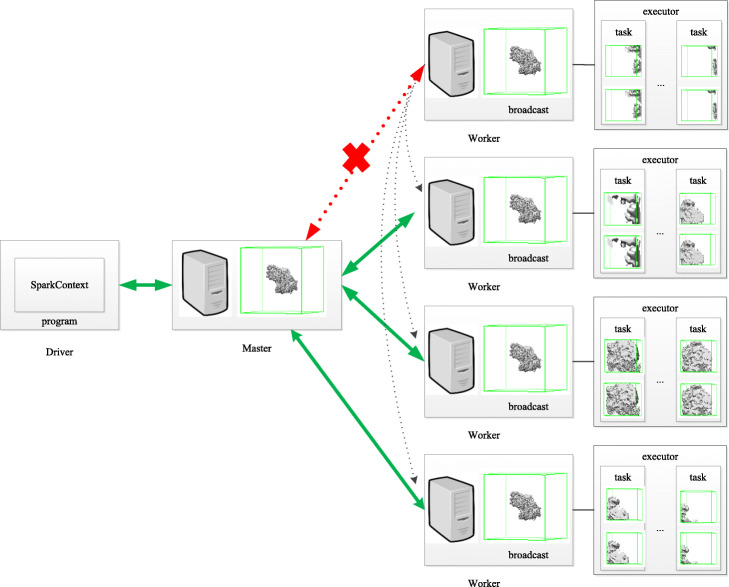
Spark-based parallel calculating 3D local FSC for 3D local resolution estimation work flow. The green arrow indicates the normal transmission and distribution of data. The red arrow indicates that the Worker node cannot connect to the Master node. The gray dotted arrow indicates that the tasks originally assigned to the crash node are reassigned to other Worker nodes

The work flow for evaluation of 3D local resolution on Spark framework is shown in Fig. 4. Our Spark-based parallel computing algorithm adopts the standalone cluster mode. The cluster manager is the Master. The Master is responsible for allocating resources, the Worker is responsible for monitoring the memory, CPU and other conditions of its own node and reporting to the Master. The Master allocates resources to Workers and monitors the resource status of the Workers at any time. Program Spark, package submitted to the Driver side, constitutes a Driver. When Spark program runs, the Driver requires resources from the Master. All tasks will be calculated in parallel by the Workers node and the results will be returned to the Master by K-V format.

### K-V format based basic fine-grained 3D array partition algorithm in Spark for parallel calculating 3D local resolution

To get the 3D local resolution map faster, 3D array partition is a key factor. K-V format based fine-grained 3D array partition algorithm theoretically confirms the maximum number of parallel execution of the algorithm. Therefore we design a K-V format based basic 3D array partition algorithm for parallel calculating 3D local FSC and estimating 3D local resolution map. The K-V format based basic 3D array partition algorithm in Spark is as follows:

Step 1: Each voxel of 3D array (3D volume density map) is transformed quadruple block (QB =(x,y,z,value)) in list.

Step 2: List containing quadruple block are transformed RDD.

Step 3: All data in RDD are added key in order to get correct partition, such as <key, QB >.

Step 4: Two RDDs are run two times operation of mapPartition and partionBy for getting the max parallel number.

Step 5: Two RDDs which should have the same number of partitions and the same number of elements in each partition, are zipped as new RDD.

Step 6: Data in each partition are transformed two 3D array, which is calculated for 3D local FSC and 3D local resolution.

Step 7: The FSC calculation results of each pair of 3D sub-volumes are filled into corresponding coordinates of the new volume, and the coordinates without filling values in the new volume is interpolated by cubic interpolation algorithm.

Here, since data in RDD is K-V pair format, in which condition all data can realize partition, 3D volume density map (3D array) must be transformed into a list of K-V format. In order to gain K-V format, all voxels in 3D volume density map must be transformed quadruple block, such as (x,y,z,v), where x, y, z is coordinate of the voxel, v is value of voxel. Then all voxels become quadruple block in list.

**Fig. 5:**
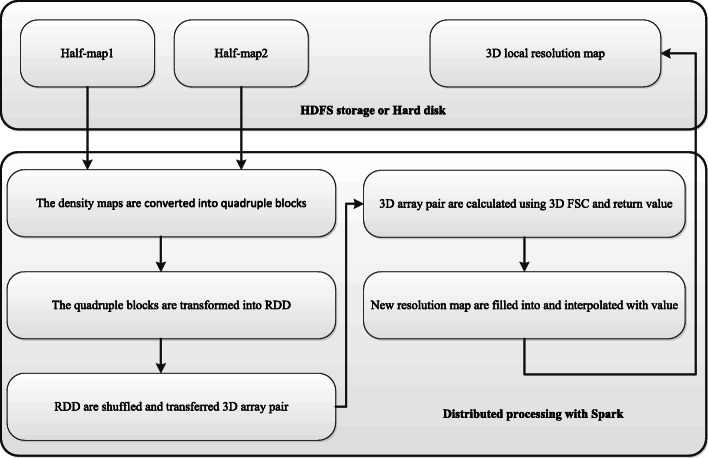
The workflow of K-V format based basic fine-grained 3D array partition algorithm in Spark for 3D local resolution estimation. Two 3D density maps are transformed RDD in Spark. K-V data in RDD are shuffled and transferred, data in each partition are transformed 3D array, which is calculated for 3D local resolution using 3D FSC. A resolution map is filled into and interpolated with calculation results according to corresponding coordinate

We show the K-V format based basic 3D array partition work flow for 3D local resolution estimation in Fig. 5. Our K-V format based basic 3D array partition algorithm in Spark requires that all values of the 3D array participate in each RDD operation, so these consume a large amount of memory space.

**Fig. 6:**
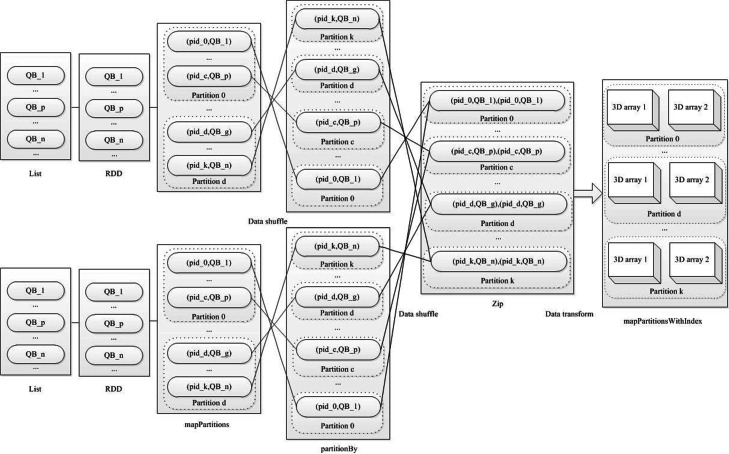
The K-V format based basic 3D array partition algorithm for 3D local resolution estimation in Spark. QB stand for quadruple block, QB=(x,y,z,v), where x, y, z is coordinate, v is voxel value. In the figure, n is volume side length, 0, c, d, k are partition number, and 0 ≤ c ≤ d ≤ k, k is the largest partition number. 3D array partition are realized by K-V data shuffle and transformation in Spark. Each 3D array pair are calculated for 3D local resolution estimation

The details of the K-V format based basic fine-grained 3D array partition algorithm in Spark are shown in Fig. 6. Values of 3D array (voxels) transforming into quadruple block are a pivotal part of 3D array partition. When 3D array becomes a list of quadruple block, the 3D array partition algorithm can exploit key and quadruple block (e.g. <key, value > pair) to operate data shuffle and partition. Finally data in each partition is converted into 3D arrays again to realize 3D array partition. In each pair 3D array, the FSC value and local resolution value are calculated, and the local resolution value is filled into corresponding coordinates of 3D local resolution map.

### K-V format based optimized fine-grained 3D array partition algorithm in Spark for parallel calculating 3D local resolution

**Fig. 7:**
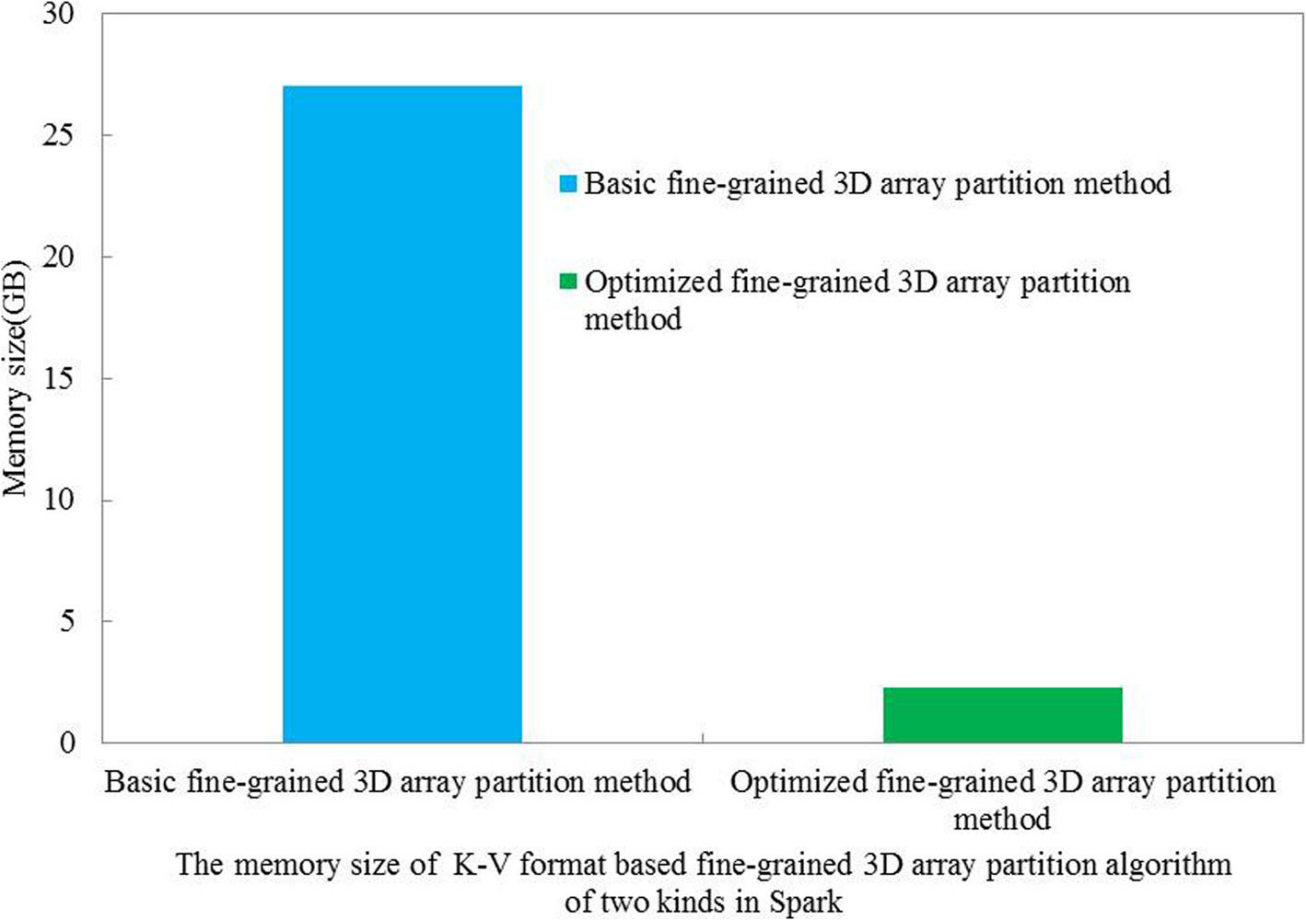
For 3D local resolution evaluation, the memory size of K-V format based basic and optimized fine-grained 3D array partition algorithm in spark

Although the K-V format based basic fine-grained 3D array partition algorithm can achieve maximum parallelization in Spark, it incurs plenty of data shuffle and transfer. So the K-V format based basic fine-grained 3D array partition algorithm results in data redundancy and consumes a large memory space in each computing node (Fig. 7). Those actions lower parallel performance in Spark. So we conceive a K-V format based optimized fine-grained 3D array partition algorithm in Spark for calculating 3D local resolution. The K-V format based optimized fine-grained 3D array partition algorithm can reduce data redundancy and shuffle in Spark.

First, the K-V format based optimized fine-grained 3D array partition algorithm in Spark reads two density map into memory; second, it gains all start coordinates of window traversing density map, and transforms K-V pair in a list; third, it transforms the list into RDD, and partition RDD; fourth, for each partition, it acquires 3D sub-array pair from 3D array, and calculates FSC and resolution with two sub-array.

From the Algorithm 1, the optimized fine-grained 3D array partition method is more concise than the basic fine-grained 3D array partition method in K-V format of Spark. The K-V format optimized fine-grained 3D array partition method in Spark doesn’t transform values of 3D array into RDD. Each RDD only contains start coordinates of partition array relative to original 3D array.

**Fig. 8:**
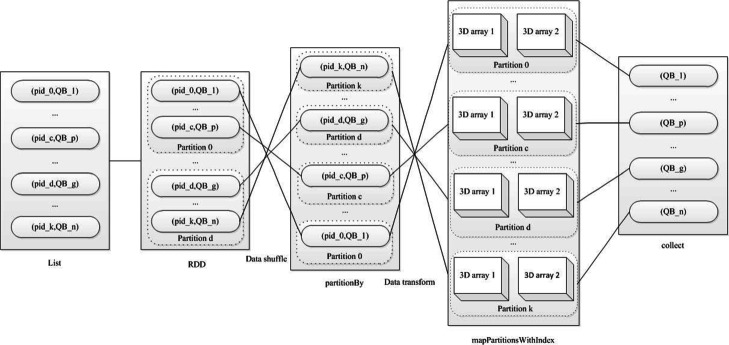
The K-V format based optimized fine-grained 3D array partition algorithm in Spark for 3D local resolution estimation. TB stand for triple block, TB=(x,y,z), where x, y, z is start coordinate of window traversing density map. 0, c, d, k are partition number, and 0 ≤ c ≤ d ≤k, k is the largest partition number. List of sequence numbering and start coordinates of window traversing are transformed into RDD. 3D sub-array pair are acquired by cutting 3D array using each partition data and side length of window, and calculated for gaining 3D local FSC and resolution

The details of the K-V format based optimized fine-grained 3D array partition algorithm in Spark are shown in Fig. 8. The total operation of the K-V format based optimized fine-grained 3D array partition algorithm is much less than that of the K-V format based basic fine-grained 3D array partition algorithm, and each RDD does not include the coordinate and values of 3D array. The two 3D arrays are cut for gaining 3D sub-array in the final operation.

The K-V format based optimized fine-grained 3D array partition algorithm in Spark highlights mainly as follows:

∙ Reduce total memory space

∙ Lessen data shuffle and transfer

∙ Enlarge parallel number

Relative to the K-V format based basic fine-grained 3D array partition algorithm in Spark, the K-V format based optimized fine-grained 3D array partition algorithm does not require many operations of data shuffle, reduces data redundancy and only needs limited memory in Spark. Hence it calculates all the tasks quickly. So we choose the K-V format based optimized fine-grained 3D array partition algorithm in Spark as the Spark-based optimized 3D local resolution estimation algorithm.

### Implementation

In this paper, the 3D local resolution evaluation based on fine-grained 3D array partition method with K-V format of Spark has been implemented in Python and C++ language, and referred to PyTom [28] in part. The input parameters of 3D local resolution evaluation algorithm based on K-V fine-grained 3D array partition in Spark mainly include two half-maps, windows side length, step size, FSC threshold, and global resolution. To further reduce the computing time, a mask and no-zeros edge length can be specified, which are optional. The global resolution value is order to calibrate the average value of all local resolution on underestimating resolution. The output is a 3D local resolution map. The 3D local resolution map can be colored with final reconstruction density map to visualize representation of reconstruction density map, where subtle change of local detail of density map can be inspected during homogeneity or heterogeneity complex reconstruction. The color tool used is Chimera [29].

Our Spark-based optimized 3D local resolution estimation code can be executed in a distributed cluster containing eight compute nodes. Configurations of each compute node include, two 1.70GHz Intel Xeon Bronze 3104 CPUs with 12 physical cores, one Gigabi ethernet card and 30GB Random Access Memory (RAM).

Deployment of Spark cluster adopt stand alone mode comprising a Master node and 4 or 8 Worker nodes. Master node mainly assigns application to Work node and maintains the status of Worker node, Driver and application. Worker nodes mainly execute tasks assigned by Master node. Our code is implemented in Spark 2.0 and Python 2.7.14.

### Experimental datasets

The two experimental density maps were acquired from the Electron Microscopy Data Bank (EMDB) to calculate 3D local resolution respectively.

The first experimental density map is the structure of human transcription factor IIH (EMDB: EMD-3802) [26], which have two half maps (single particle analysis map from independently aligned half dataset 1 and 2), the resolution is 4.4Å, FSC threshold is 0.143, the number of grid points is 256×256×256, the voxel size is 1.32×1.32×1.32Å.

The second experimental density map is the structure of SA of Sar1-Sec23-Sec24 (EMDB: EMD-0044) [27], which have two half maps (average map from independently aligned half dataset 1 and 2), the resolution is 4.9Å, FSC threshold is 0.143, the number of grid points is 224×224×224, the voxel size is 1.33×1.33×1.33Å.

## Data Availability

Two half maps of structure of human transcription factor IIH (EMDB: EMD-3802) can be downloaded at https://www.emdataresource.org/EMD-3802 Two half maps of SA of Sar1-Sec23-Sec24 (EMDB: EMD-0044) can be downloaded at https://www.emdataresource.org/EMD-0044 The source code of the Spark-based parallel calculation of 3D Fourier shell correlation for macromolecule structure local resolution estimation algorithm is available at https://github.com/xulabs/projects.

## Abbreviations

cryo-EM: cryoelectron Microscopy
SPA: Single particle analysis
SA: Subtomogram averaging
cryo-ET: Electron cryotomography
FSC: Fourier shell correlation
K-V: Key-value data
SSNR: Spectral signal-to-noise ratio
OpenMP: Open multi-processing
SNR: Signal-to-noise ratio
MPI: Message passing interface
RDD: Resilient distributed datasets
API: Application programming interface
EMDB: Electron microscopy data bank
GPU: Graphics processing unit
RAM: Random access memory
